# Multimodal techniques for maximal safe resection of IDH-mutant low-grade glioma involving corpus callosum, a retrospective study and prognosis analysis

**DOI:** 10.1186/s41016-026-00432-y

**Published:** 2026-05-01

**Authors:** Meng Cui, Meng Zhang, Chunhui Zhou, Guochen Sun, Jianning Zhang

**Affiliations:** 1 The Sixth Medical Center, Chinese People’s Liberation Army General Hospital, Beijing, China; 2 Senior Department of Neurosurgery, Chinese People’s Liberation Army General Hospital, Beijing, China

**Keywords:** Low-grade glioma, Corpus callosum, Neuronavigation, Intraoperative magnetic resonance, Intraoperative neuromonitoring, Multimodal techniques

## Abstract

**Background:**

The IDH-mutant low-grade glioma (LGG) involving corpus callosum (ccLGG) is a rare type of LGG which has a poorer prognosis. To evaluate the efficacy of multimodal techniques (comprising neuronavigation, intraoperative MRI, and neuromonitoring) compared with the conventional approach guided solely by neuronavigation in the resection of IDH-mutant ccLGG, and to identify prognostic factors of ccLGG.

**Methods:**

The IDH-mutant ccLGG cases that received resection in our center between 2014 and 2022 were collected and reviewed retrospectively. Comparisons were made between the multimodal and conventional groups regarding patient demographics, tumor characteristics, extent of resection (EOR), neurological function, Karnofsky Performance Status (KPS), progression-free survival (PFS), and overall survival (OS).

Both univariate and multivariate analysis were employed to assess potential prognostic factors.

**Results:**

Ultimately, 64 patients in the multimodal group and 34 in the conventional group were enrolled. Compared with the conventional group, the multimodal group achieved a significantly higher median EOR (100% vs. 93.55%, *P* = 0.001) and a greater gross total resection (GTR) rate (62.5% vs. 35.3%, *P* = 0.010). No significant differences were observed in postoperative neurological function or KPS between the two groups at any of the time points assessed. Compared with the conventional group, the multimodal group demonstrated significantly longer median PFS (78.5 vs. 48.1 months, *P* = 0.010) and OS (106.1 vs. 66.9 months, *P* = 0.009). Non-butterfly ccLGG, less tumor volume, genu invasion of CC (compared to splenium invasion), less volume of CC invasion, higher EOR, longer chemotherapy cycles of Temozolomide (TMZ), higher KPS on 3 months and MGMT methylation were positive factors for PFS of ccLGG. While four factors were associated with the longer OS of ccLGG, including genu of CC invasion compared to both genu and body invasion of CC, higher EOR, longer TMZ cycles and MGMT methylation.

**Conclusion:**

Multimodal techniques are useful for achieving maximal safe resection and better prognosis of ccLGG. As higher EOR independently predicts improved survival in IDH-mutant ccLGG, maximal safe resection should be suggested and achieved by appropriate means tailored to available resources. The postoperative chemotherapy should also be recommended to patients with high risks. While whether postoperative radiotherapy should be performed should consider and balance many factors.

**Supplementary Information:**

The online version contains supplementary material available at 10.1186/s41016-026-00432-y.

## Background

Low-grade glioma (LGG) accounts for about 25% of all gliomas [1, 2]. The 2021 WHO classification of tumors of the central nervous system considered glioma of WHO grade 1 and 2 as LGG, and IDH mutation is one of typical molecular characteristics in astrocytoma and oligodendroglioma of WHO grade 2 [3]. The current standard treatment of IDH-mutant LGG involves maximal safe resection followed by postoperative radiochemotherapy, particularly for high-risk patients [4]. A distinct subset of LGGs that poses challenges for maximal safe resection and comprehensive oncologic management consists of those infiltrating the corpus callosum. Consequently, corpus callosum infiltrating LGGs (ccLGGs) are associated with a less favorable prognosis compared to LGGs without corpus callosum involvement [5–7]. This subset can be further categorized into non-butterfly ccLGGs, where the lesion infiltrates one side of the cerebral hemisphere, and butterfly lesions (bLGGs), involving both cerebral hemispheres and the corpus callosum [8–10]. These anatomic distinctions (butterfly versus non-butterfly) are imperative for ensuring a nuanced and meaningful survival analysis of ccLGG patients.

Recent advancements in multi-modal intraoperative techniques, specifically intraoperative MRI (iMRI) aided neuronavigation and intraoperative neuromonitoring (IONM), have significantly elevated the capacity for achieving maximal safe resection of ccLGGs. Our institution embraced this multi-modal paradigm by installing an iMRI in 2009. During this transition, certain ccLGG cases did not undergo multi-modality aided resection due to resource constraints, such as the availability of the IONM team or iMRI. Despite these limitations, all surgeries were performed by the same surgical team, with the goal of achieving maximal safe resection. Here, we conducted a comparative analysis of the extent of resection (EOR) and survival with or without multimodality-aided surgery for IDH-mutant ccLGG patients. Prognostic factors were also explored in these patients.

## Methods

### Patient selection

Clinical data of glioma patients were retrospectively collected from electronic medical records (EMRs) in the Department of Neurosurgery at our hospital from January 2014 to December 2022. The end time point of follow-up was May 2025. The decision of using multimodal techniques or conventional approach intraoperatively was made by both surgeon and patients (their relatives) based on patients’ conditions and tumor characteristics. Approval for the study was granted by our institutional ethics committee (No. S2024-098-01). All patients or their relatives had previously provided written informed consent for surgery. Eligible patients with ccLGG were identified from the EMRs according to the following criteria: (1) surgery performed in our department, (2) pathologically and genetically confirmed IDH-mutant LGG of WHO grade 2, (3) preoperative MRI evidence of corpus callosum invasion, and (4) treatment with multimodal techniques or the conventional approach. Exclusion criteria included (1) isolated lesion of the CC, (2) patients who underwent biopsy only, (3) additional isolated lesions other than ccLGG, and (4) patients lost to follow‑up.

### Patient groupings

Based on the surgical approach, patients with IDH-mutant ccLGG were categorized into two groups: the multimodal group, in which surgery was assisted by neuronavigation, intraoperative MRI, and neuromonitoring, and the conventional group, in which surgery was guided solely by neuronavigation. The multimodal group was further divided into the subgroups of bLGG group and non-butterfly ccLGG group.

### Clinical variables

Preoperative variables—including age, sex, symptoms, muscle strength (Medical Research Council scale), aphasia quotient (AQ, Western Aphasia Battery) [11, 12], cognitive function (Montreal Cognitive Assessment (MoCA) scale), and KPS—were recorded. Tumor-related variables included tumor location, volume, histopathology, MGMT methylation status, combined deletions of chromosome 1p and 19q. Treatment-related variables consisted of radiotherapy and the number of temozolomide (TMZ) chemotherapy cycles. Postoperative outcome variables comprised surgery time, length of hospital stay, EOR, muscle strength, AQ, MoCA, KPS, incidences of surgery-related complications (including ischemia, hemorrhage, and edema), incidences of neurological deficits, as well as progression-free survival (PFS) and overall survival (OS).

### Image acquisition and volumetric analysis

Preoperative MRI was acquired using a 3.0 Tesla scanner (Siemens Espree, Erlangen, Germany) for all patients. The scanning sequences were consistent with our previous study [13]. When the tumor invaded or was adjacent to eloquent areas, blood oxygen level-dependent functional MRI and diffusion tensor imaging were performed to identify eloquent cortices and fiber tracts. Intraoperative MRI or postoperative MRI within 48 h after surgery was used to evaluate residual tumor and EOR. Digital data from all MRI sequences were transferred to iPlan software 2.6 (Brainlab Feldkirchen, Germany). Manual delineation of a region of interest (ROI) that included the tumor and the invaded corpus callosum was performed using the object creation module in the program. Tumor volumes (cm^3^) were automatically calculated pre- and postoperatively via the software on the basis of tumor tissue on T2 or FLAIR images, and gross total resection (GTR) was defined as achieving an EOR of 100% [6, 14].

### Surgical approaches and techniques

In an iMRI-compatible operating room, all surgeries utilized neuronavigation. Following craniotomy and dural opening, the surgeon resected the tumor under neuronavigation guidance, with navigation information available both on the monitor and within the microscope view. In cases of bLGG with bilateral brain lobes involvement, the surgical approach prioritized protection of functional cortex and tracts by beginning tumor removal from the hemisphere bearing the larger tumor burden using a transcortical route. If the tumor volumes were comparable on both sides, the nondominant hemisphere was chosen to create the resection corridor for performing corticectomy. After the ipsilateral tumor was totally resected, the contralateral tumor was accessed and removed through the longitudinal fissure. The majority of tumors were located and resected below the falx cerebri; occasionally, partial falx resection was performed to enhance exposure of the contralateral lesion [13]. By creating a corridor along the medial surface of the brain through the longitudinal fissure, the contralateral tumor—including distal portions—was clearly visualized and resected, allowing preservation of the contralateral cortices. When the cingulate gyrus was involved by the tumor, it was sacrificed to achieve maximal resection. In cases without cingulate gyrus invasion, the contralateral tumor was exposed and removed from above and below the cingulate gyrus, enabling the resection to converge and the cingulate gyrus to remain intact. Critical anatomical structures, such as the basal ganglia and important vessels, were visualized and protected during surgery.

**Fig. 1:**
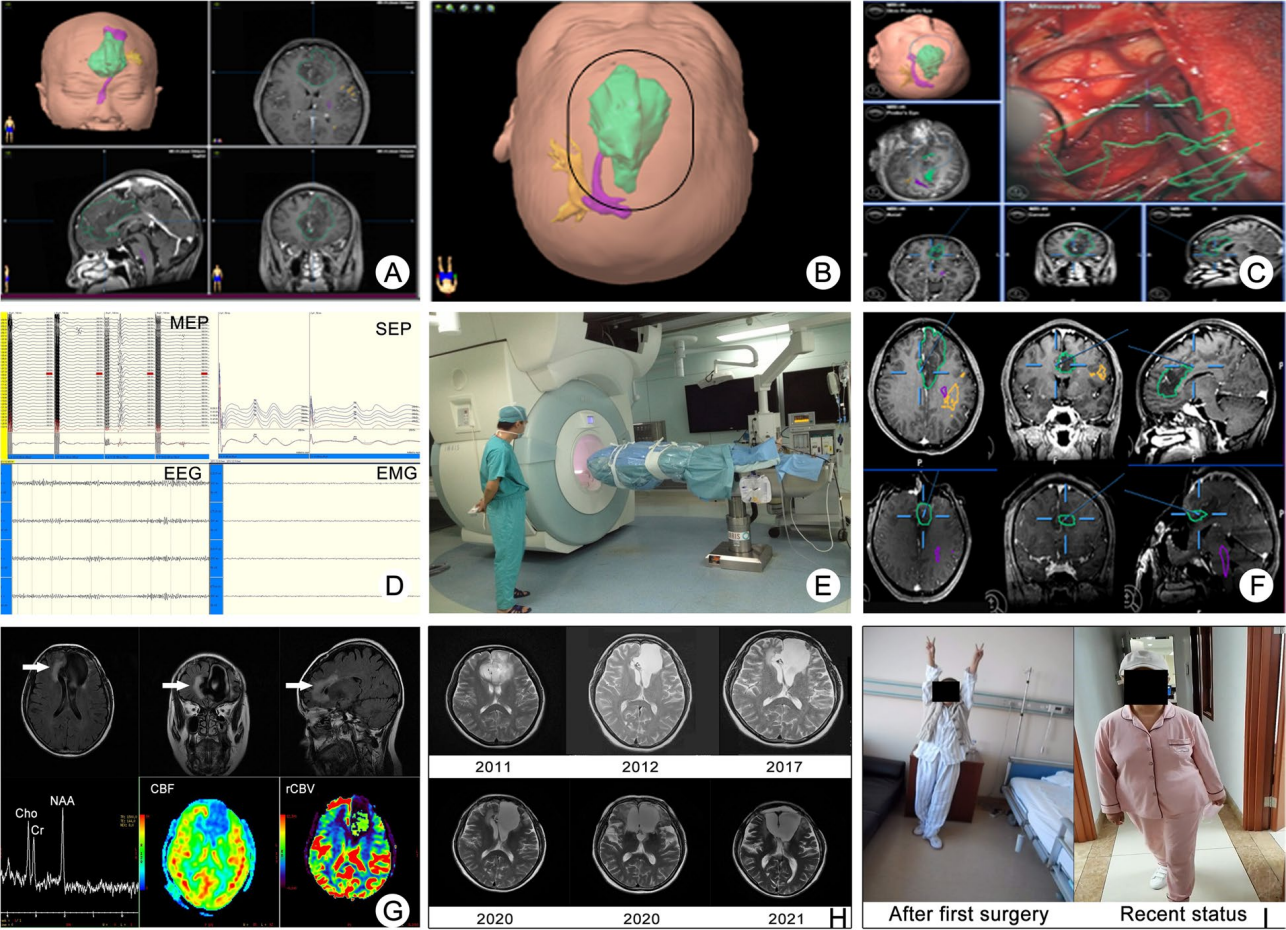
A case of bLGG resection assisted by multimodal techniques. This patient was a 49-year-old woman with no preoperative symptoms and a KPS of 100. The final pathological diagnosis was oligodendroglioma, WHO grade 2. **A** Preoperative plan of neuronavigation designed by the surgeon, pyramidal tract (purple), arcuate fasciculus (yellow) and tumor (green). **B** Bilateral minimally invasive craniotomy was designed to avoid injury to the superior sagittal sinus. **C** Resection guided by neuronavigation on a screen and under a microscope. **D** Continuous MEPs, SEPs, EEG and EMG monitoring, as well as their alterations during the resection process. **E** iMRI magnet moved to the OR for scanning. **F** Residual tumor was assessed on iMRI images. According to the iMRI DICOM data, the neuronavigation can be updated to perform further resection. **G** Her neurological function was normal all the time after surgery, no other symptoms and complications occurred. The patient was performed radiotherapy and 12 cycles of TMZ chemotherapy. During the follow-up, the multimodal MRI showed that she had a suspicious tumor recurrence in right frontal lobe close to the edge of resection cavity. She received the second surgery assisted by multimodal approach on 99.6 months after the first surgery. The second pathological diagnosis was astrocytoma, WHO grade 2. **H** The long-term follow-up of this patient. She only had a mild memory loss at present with a KPS of 90. **I** The functional status of this patient after first surgery and recently

For tumors adjacent to eloquent areas, awake craniotomy and iIONM were performed using the Endeavor CR system (Nicolet®, USA). Direct electrical stimulation with a monopolar probe was used to map functional cortex and tracts, while a neurophysiologist continuously monitored neurological function and evoked potentials throughout the procedure [15]. Upon the surgeon’s assessment that the tumor had been fully removed, sterile drapes were placed over the surgical field. The iMRI magnet was subsequently positioned semiautomatically for MRI scanning. In cases where residual tumor was detected on T2 or FLAIR images, the iMRI data were transferred to iPlan software to update the surgical plan [16]. Then, the surgeon continued to remove the residual tumor (Fig. 1).

All high-risks patients with ccLGG were advised to receive radiotherapy and adjuvant TMZ chemotherapy. The high risks included age≧ 60 years, not gross total resected, tumor size > 6 cm in diameter and neurological defects before surgery [4, 17]. Follow-up, including MRI scans, was performed every three months for the patients.

### Statistical analysis

Statistical analysis were carried out with SPSS 21.0 software. Continuous parametric variables were compared between groups using Student’s *t*-test, categorical variables were analyzed using the *χ*^2^ test (or Fisher’s exact test where applicable), and the Mann–Whitney *U* test was applied for continuous nonparametric variables. The Kaplan–Meier method was used to estimate survival curves, which were subsequently compared using the log-rank test. To identify independent prognostic factors for PFS and OS, univariate and multivariate analyses were performed using Cox proportional hazards models. Statistical significance was set at *P* < 0.05. The meta-analysis of incidences of samples among previous studies was performed by STATA 16.0. Heterogeneity among studies was assessed using the Q test and the inconsistency index (*I*^2^). Significant heterogeneity was defined as a *Q* test *P* value < 0.1 or *I*^2^ > 50%, in which case the incidence was pooled by a random‑effects model. Otherwise, a fixed‑effects model was applied.

## Results

**Fig. 2:**
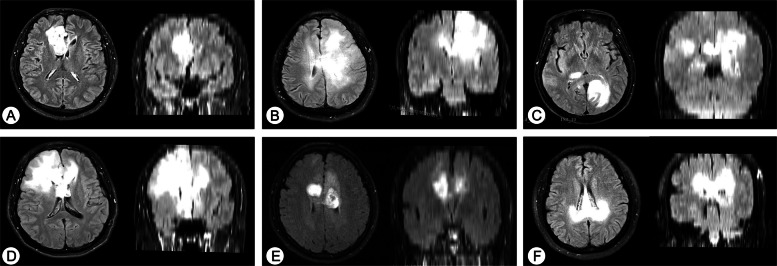
Character of ccLGG that invaded different parts of corpus callosum. All images originated from axial and coronal T2Flair sequence. Genu invasion of CC (**A**, **D**), body invasion of CC (**B**, **E**), and splenium invasion of CC (**C**, **F**). The upper column shows the non-butterfly ccLGG (single-wing butterfly type, **A**–**C**), the lower column shows the bLGG (**D**–**F**)

Among 796 patients with supratentorial LGG, 109 of them had IDH-mutant ccLGG, accounting for 13.7% of LGG. Among all the 109 patients with ccLGG, 46 cases (42.2%) had bLGG and 63 cases (57.8%) non-butterfly ccLGG. Figure 2 presented the typical morphological characteristics of bLGG and non-butterfly ccLGG and the Supplementary Fig. S1 showed their postoperative MRI. For the 109 patients, seven patients who underwent biopsy only, 1 patient who had isolated lesion of corpus callosum, and 3 patients who were lost to follow-up were excluded. Finally, 98 patients of IDH-mutant ccLGG that underwent resection were included, and 64 cases (24 bLGG and 40 non-butterfly ccLGG) were in multimodal group and 34 cases (16 bLGG and 18 non-butterfly ccLGG) were in conventional group.

### Baseline characteristics

The preoperative baseline characteristics of multimodal and conventional groups were summarized in Table 1. There were no significant differences in baseline characteristics between the two groups.

**Table 1:** Baseline clinical and tumor characteristics between the multimodal and conventional group

Variables	Multimodal group (*N* = 64)	Conventional group (*N* = 34)	*P*
Age (years, x̄ ± sd)^†^	42.7 ± 11.5	43.6 ± 12.5	0.72
Sex (*N* [%])			0.41
Male	32 (50.0)	20 (58.8)	
Female	32 (50.0)	14 (41.2)	
Muscle strength			
Grades (0–5 level)^‡^	4.89 ± 0.22	4.86 ± 0.25	0.45
Language			
AQ‡	96.0 ± 10.0	96.2 ± 10.6	0.70
Cognitive impairment			
Median MoCA score (IQR)^‡^	30 (26.25–30)	30 (26–30)	0.93
Median preop. KPS (IQR)^‡^	80 (70–90)	80 (70–90)	0.40
Other symptoms (*N* [%])			
Headache	28 (43.8)	20 (58.8)	0.16
Dizziness	14 (21.9)	4 (11.8)	0.22
Seizure	27 (42.2)	12 (35.3)	0.51
Nausea/vomiting	13 (20.3)	8 (23.5)	0.71
Hypoesthesia	6 (9.4)	2 (5.9)	0.83
Vision defect	4 (6.3)	1 (5.9)	0.82
bLGG (*N* %)	24 (37.5)	16 (47.1)	0.36
Non-butterfly ccLGG (*N* [%])	40 (62.5)	18 (52.9)	
Tumor location (*N* [%])			0.12
Frontal lobe involved alone	42 (65.6)	18 (52.9)	
Insular/temporal lobe involved	14 (21.9)	6 (17.6)	
Parietal/parietooccipital lobe involved	8 (12.5)	10 (29.4)	
Site of CC invasion (*N* [%])			0.62
Genu	38 (59.4)	15 (44.1)	
Body	10 (15.6)	7 (20.6)	
Splenium	6 (9.4)	4 (11.8)	
Genu and body	6 (9.4)	6 (17.6)	
Splenium and body	4 (6.3)	2 (5.9)	
Preop. Total tumor Vol (cm^3^, median [IQR])‡	65.45 (43.51–90.34)	62.66 (44.15–110.86)	0.90
CC invasion Vol (cm^3^, median [IQR])^‡^	4.62 (3.83–6.86)	5.31 (3.71–7.90)	0.36
Ratio of CC invasion/Total Vol (%, median [IQR])^‡^	7.14 (4.91–11.15)	8.37 (5.09–12.12)	0.49
High risks (*N* [%])	53 (82.8)	29 (85.3)	0.75
MGMT methylation (*N* [%])	42 (65.6)	17 (50.0)	0.13
Pathology (*N* [%])			0.89
Oligodendroglioma	31 (48.4)	16 (47.1)	
Astrocytoma	33 (51.6)	18 (52.9)	
Radiotherapy (*N* [%])	47 (73.4)	21 (61.8)	0.23
TMZ chemotherapy (*N* [%])	56 (87.5)	27 (79.4)	0.29
Median TMZ cycles (IQR)^‡^	8 (6–15)	6 (2.75–13.5)	0.12

*IQR* interquartile range

Bold face type indicates statistical significance

^†^Calculated by independent samples *t* test

^‡^Calculated by Mann–Whitney *U*-test

### Outcomes and survival

**Fig. 3:**
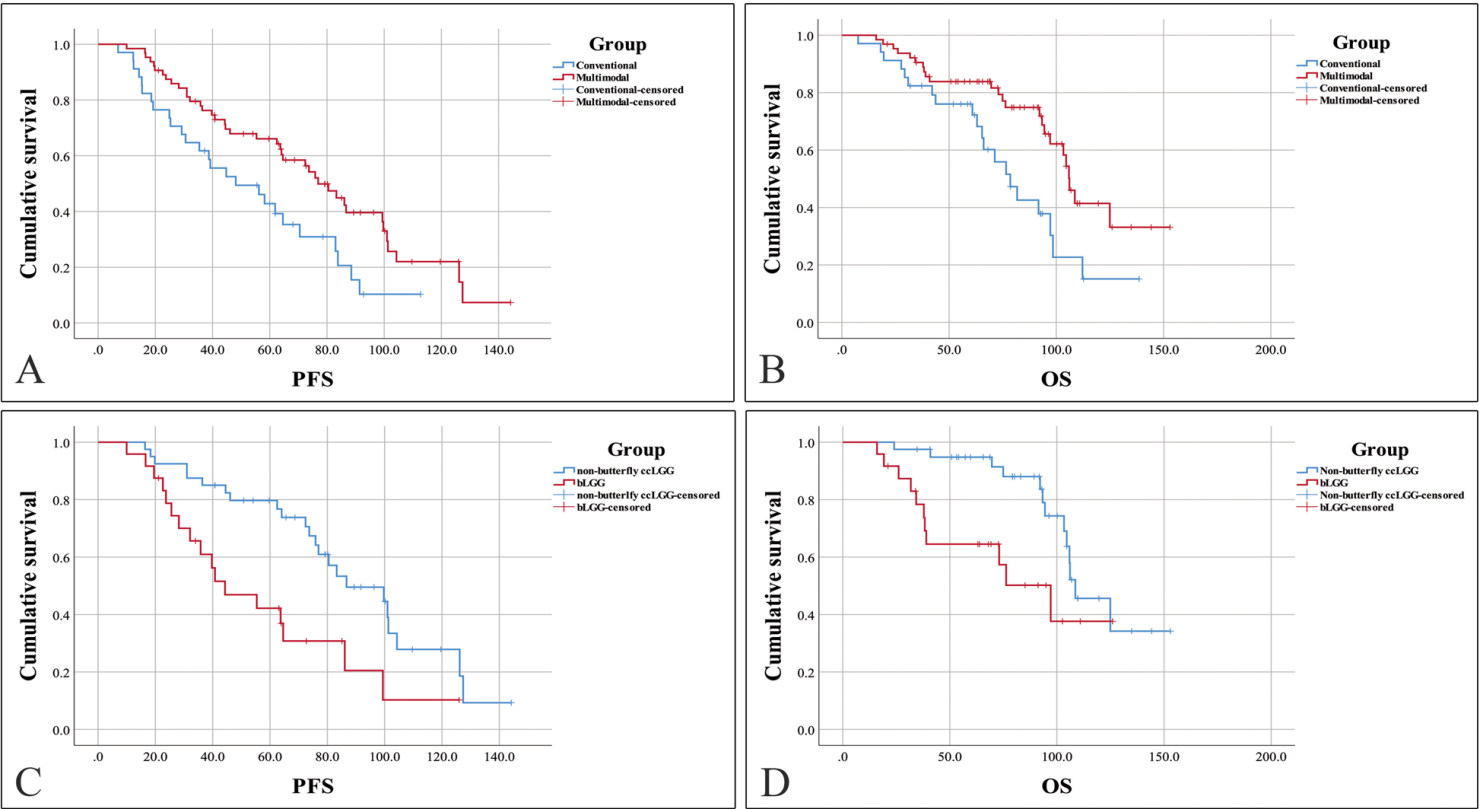
Survival curves of different groups. Multimodal group versus conventional group (**A**, **B**), bLGG versus non-butterfly ccLGG (**C**, **D**)

The patients in multimodal group achieved a higher median EOR (100% [IQR 96.10–100% versus 93.55% [IQR 84.89–100%], *P* = 0.001) than the patients in conventional group (Table 2). The patients in multimodal group also had the higher rate of GTR (62.5% versus 35.3%, *P* = 0.010). While the median surgery time was not different between two groups (7.17 versus 7.21 h, *P* = 0.69), and the median length of hospital stay was also not different (19 versus 18 days, *P* = 0.30). The patients’ muscle strength, AQ, MoCA score, and KPS were not different between the multimodal and conventional groups at different time points of follow-up. Compared to the preoperative KPS, the postoperative KPS improved significantly on 3 months after surgery in both multimodal group (*P* = 0.001) and conventional group (*P* = 0.013). The survival analysis indicated that the patients in multimodal group had longer median PFS (78.5 versus 48.1 months, *P* = 0.010) than the patients in conventional group. The median OS of the patients in multimodal group was also longer than the patients in conventional group (106.1 versus 66.9 months, *P* = 0.009) (Fig. 3A, B). However, the 5-year survival rates of two groups were not different significantly (84.4% versus 76.5%, *P* = 0.34). In the multimodal group, patients diagnosed with astrocytoma had shorter PFS and OS than those diagnosed with oligodendroglioma (Supplementary Fig. S2A, B). While in the conventional group, the PFS and OS were both not significantly different among patients diagnosed either with astrocytoma or oligodendroglioma (Supplementary Fig. S2C, D).

**Table 2:** Outcomes of multimodal group and conventional group

Variables	Multimodal group (64)		Conventional group (34)			P*
Median length of hospital stay (IQR)^‡^	19 (16–22)		18 (15–20.25)			0.30
Median surgery time (IQR)^‡^	7.17 (6.54–7.98)		7.21 (6.49–8.02)			0.69
Other surgery-related complications (*N* [%])	0 (0)		2 (5.9)			/
Median EOR (% [IQR])	100 (96.10–100)		93.55 (84.89–100)			**0.001**
Rate of GTR (*N* [%])	40 (62.5)		12 (35.3)			**0.010**
Muscle strength (x̄ ± sd)^‡^						
Preoperative	4.89 ± 0.22	P#	4.86 ± 0.25		P#	0.45
At discharge	4.80 ± 0.42	0.35	4.74 ± 0.39		0.33	0.38
Postoperative 3 months	4.89 ± 0.27	0.72	4.84 ± 0.30		0.93	0.27
Permanent deficits (*N* [%])^§^	3 (4.7)		6 (17.6)			0.08
AQ (x̄ ± sd)‡						
Preoperative	96.0 ± 10.0	P#	96.2 ± 10.6		P#	0.70
At discharge	95.6 ± 8.9	0.57	94.8 ± 12.8		0.54	0.85
Postoperative 3 months	97.0 ± 6.8	0.86	96.8 ± 7.4		0.66	0.99
Permanent deficits (*N* [%])^§^	4 (6.3)		4 (11.8)			0.57
Median MoCA score (IQR)‡						
Preoperative	30 (26.25–30)	P#	30 (26–30)		P#	0.93
At discharge	30 (26–30)	0.62	30 (26–30)		0.57	0.77
Postoperative 3 months	30 (28–30)	0.76	30 (26–30)		0.71	0.54
Permanent deficits (*N* [%])^§^	6 (9.4)		3 (8.8)			0.93
Median KPS (IQR)^‡^						
Preoperative	80 (70–90)	P#	80 (70–90)		P#	0.40
At discharge	80 (70–97.5)	0.39	90 (60–90)		0.46	0.48
Postoperative 3 months	90 (80–97.5)	**0.001**	90 (77.5–100)	**0.013**		0.84
Median PFS in months (95% CI)^‡^	78.5 (63.5–93.5)		48.1 (22.8–73.4)			**0.010**
Median OS in months (95% CI)^‡^	106.1 (100.6–111.6)		66.9 (52.9–80.9)			**0.009**
5-year survival (*N* [%])	54 (84.4)		26 (76.5)			0.34

*IQR* interquartile range, *sd* standard deviation

Bold face type indicates statistical significance

*Comparison between the multimodal group and conventional group

^#^Compared the preoperative and postoperative neurological function

^‡^Calculated by Mann–Whitney *U* test

^§^Cases that had permanent neurological deficits divided by total cases

### Residual tumors on iMRI and postoperative MRI

**Fig. 4:**
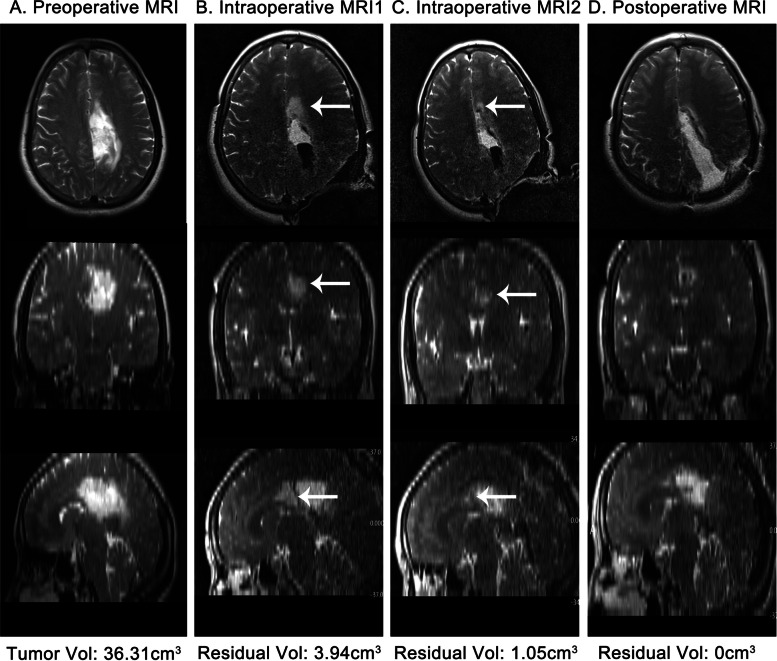
Multiple use of iMRI increasing EOR of ccLGG. This case was a 58-year-old woman with a pathological diagnosis of oligodendroglioma (WHO grade 2). Pre- (**A**), intra- (**B**, **C**), and post (**D**) MRIs showed the residual tumor and further resection, which increased the EOR from initial 89.15% to final 100%. The tumor volume was measured by Brainlab automatically. The white arrows showed the residual tumor was located in the body of CC (the most anterior part of tumor) close to motor tract

Residual tumors were identified in 46 patients on iMRI or postoperative MRI. Residual tumors were often located in eloquent areas (67.4%), such as basal ganglia, internal capsule and thalamus, etc. The contralateral and ipsilateral corpus callosum (50.0%) and the distal part of contralateral brain lobes (13.0%) were also the predilection sites of residual tumors. In the multimodal group, ten patients had residual tumors on the first iMRI scan and then further resection was preformed (Fig. 4). The final median EOR increased from 90.34 to 100% (*P* < 0.001).

### Comparison of bLGG with non-butterfly ccLGG in multimodal group

The baseline characteristics of bLGG and non-butterfly ccLGG were not different significantly (Supplementary Table S1). By using multimodal approach, the clinical outcomes were similar between bLGG and non-butterfly ccLGG. While the survival analysis indicated that the patients with bLGG had shorter median PFS (44.3 versus 86.7 months, *P* = 0.004) and OS (97.1 versus 108.6 months, *P* = 0.019) than the patients with non-butterfly ccLGG (Fig. 3C, D). The estimated 5-year survival of the patients with bLGG was also lower than those with non-butterfly ccLGG (66.7% versus 95.0%, *P* = 0.008).

### Prognosis analysis of ccLGG

Univariate analysis demonstrated that 10 factors were significantly associated with the PFS of ccLGG, including bLGG, tumor volume, preoperative KPS, site of CC invasion, volume of CC invasion, multimodal approach, EOR, radiotherapy, TMZ cycles, KPS on 3 months and MGMT methylation (*P* < 0.1). Ten factors were significantly associated with the OS, including bLGG, tumor volume, preoperative KPS, site of CC invasion, volume of CC invasion, multimodal approach, EOR, pathology (astrocytoma versus oligodendroglioma), TMZ cycles and MGMT methylation (*P* < 0.1) (Table 3).

**Table 3:** Prognostic factors of ccLGG by univariate analysis

Factors	HR for PFS (95% CI)	*P*	HR for OS (95% CI)	*P*
Preoperative				
bLGG	2.41 (1.46–3.98)	**0.001**	1.96 (1.08–3.56)	**0.026**
Age	0.99 (0.98–1.02)	0.76	0.99 (0.97–1.02)	0.70
Sex	0.82 (0.51–1.34)	0.43	0.99 (0.55–1.79)	0.97
Tumor volume	1.01 (1.01–1.02)	**< 0.001**	1.01 (1.01–1.02)	**0.001**
Pre-KPS	0.98 (0.96–0.99)	**0.037**	0.97 (0.94–0.99)	**0.016**
Site of CC invasion (compared to Genu)		**0.007**		**0.009**
Body	1.28 (0.65–2.52)	0.48	1.44 (0.63–3.29)	0.39
Splenium	1.93 (0.85–4.38)	0.12	2.59 (0.96–6.99)	**0.06**
Genu and body	3.23 (1.60–6.52)	**0.001**	4.04 (1.71–9.53)	**0.001**
Splenium and body	3.05 (1.17–7.93)	**0.022**	3.43 (1.16–10.20)	**0.026**
CC invasion volume	1.22 (1.12–1.33)	**< 0.001**	1.14 (1.04–1.25)	**0.007**
Ratio of CC invasion/Total Vol	1.01 (0.97–1.05)	0.76	0.97 (0.92–1.03)	0.34
Intraoperative				
Multimodal approach	0.52 (0.31–0.86)	**0.011**	0.46 (0.25–0.84)	**0.011**
EOR	0.95 (0.93–0.96)	**< 0.001**	0.93 (0.92–0.95)	**< 0.001**
Postoperative				
Pathology Astrocytoma	1.38 (0.84–2.24)	0.20	1.71 (0.94–3.13)	**0.08**
Radiotherapy	0.73 (0.43–1.23)	0.24	0.65 (0.35–1.21)	0.17
TMZ cycles	0.94 (0.91–0.97)	**< 0.001**	0.92 (0.87–0.96)	**< 0.001**
KPS on 3 months	0.98 (0.97–0.99)	**0.04**	0.98 (0.97–1.00)	0.11
MGMT methylation	0.40 (0.24–0.67)	**0.001**	0.30 (0.15–0.61)	**0.001**

Multivariate analysis showed that the non-butterfly ccLGG (HR = 0.26, *P* < 0.001), less tumor volume (HR = 0.99, *P* = 0.002), genu invasion of CC (compared to splenium invasion) (HR = 0.20, *P* = 0.002), less volume of CC invasion (HR = 0.78, *P* < 0.001), higher EOR (HR = 0.95, *P* = 0.001), longer TMZ cycles (HR = 0.93, *P* = 0.001), higher KPS on 3 months (HR = 0.97, *P* = 0.009) and MGMT methylation (HR = 0.27, *P* < 0.001) were associated with the longer PFS of patients (*P* < 0.05). Multivariate analysis demonstrated that four factors were associated with the longer OS of patients, including genu of CC invasion compared to both genu and body invasion of CC (HR = 0.24, *P* = 0.016), higher EOR (HR = 0.92, *P* < 0.001), longer TMZ cycles (HR = 0.94, *P* = 0.036) and MGMT methylation (HR = 0.37, *P* = 0.029) (Table 4).

**Table 4:** Prognostic factors of ccLGG by multivariate analysis

Factors	HR for PFS (95% CI)	*P*	HR for OS (95% CI)	*P*
Preoperative				
bLGG	3.84 (1.87–7.87)	**< 0.001**	1.60 (0.73–3.48)	0.24
Tumor volume	1.01 (1.00–1.02)	**0.002**	1.01 (1.00–1.01)	0.29
Pre-KPS	1.02 (0.99–1.04)	0.28	1.00 (0.97–1.03)	0.94
Site of CC invasion (compared to Genu)		**0.005**		**0.015**
Body	0.73 (0.28–1.92)	0.53	0.38 (0.09–1.51)	0.17
Splenium	4.97 (1.77–14.01)	**0.002**	2.18 (0.66–7.22)	0.20
Genu and body	2.39 (0.94–6.08)	0.07	4.18 (1.31–13.39)	**0.016**
Splenium and body	0.81 (0.23–2.91)	0.75	1.03 (0.20–5.24)	0.97
CC invasion volume	1.28 (1.13–1.45)	**< 0.001**	1.10 (0.95–1.27)	0.22
Intraoperative				
Multimodal approach	0.65 (0.35–1.22)	0.18	0.79 (0.38–1.64)	0.52
EOR	0.95 (0.92–0.98)	**0.001**	0.92 (0.89–0.96)	**< 0.001**
Postoperative				
Pathology astrocytoma	/	/	1.53 (0.67–3.50)	0.31
TMZ cycles	0.93 (0.90–0.97)	**0.001**	0.94 (0.89–1.00)	**0.036**
KPS on 3 months	0.97 (0.95–0.99)	**0.009**	/	/
MGMT methylation	0.27 (0.14–0.51)	**< 0.001**	0.37 (0.15–0.91)	**0.029**

## Discussion

The glioma with CC invasion was considered to be associated with poorer prognosis of patients. Some previous studies had explored the maximal safe resection of this kind of glioma. But most of them only included glioblastoma especially the butterfly glioblastoma [5, 6, 13, 18–23]. Few studies explored the maximal safe resection of IDH-mutant ccLGG. In this study, we summarized our experience of maximal safe resection of IDH-mutant ccLGG by using multimodal techniques since 2014. The patients with ccLGG achieved good clinical outcomes and survival with the management by using multimodal techniques. Positive prognostic factors of ccLGG were also determined. It can be concluded that aggressive resection by using multimodal techniques followed with postoperative chemotherapy of long cycles should be recommended for all patients with bLGG or non-butterfly ccLGG involved any parts of corpus callosum.

The patients managed with multimodal approach achieved a higher EOR than with the conventional surgery without causing more surgery-related complications. The rate of GTR reached 62.5%, which was higher than that of conventional surgery (35.3%). Furthermore, compared to preoperative and conventional group’s neurological functional status, the patients treated with multimodal approach both achieved the preservation of neurological function postoperatively, including the motor, language, and cognitive function. However, both multimodal group and conventional group had the KPS improved significantly on 3months postoperatively; thus, no matter what intraoperative techniques were used, tumor resection can benefit the neurological function of ccLGG. Because of the higher EOR, the multimodal approach can significantly prolong the PFS and OS of the patients with ccLGG than the conventional resection.

The patients with bLGG in multimodal group had the similar EOR, GTR rate and postoperative KPS with the patients with non-butterfly ccLGG, which demonstrated the multimodal approach can be beneficial for the patients with both types of ccLGG. However, the survival analysis indicated that the patients with bLGG had significantly shorter PFS and OS than those with non-butterfly ccLGG. Previous studies concluded that larger tumor volume was associated with poorer survival of glioma [24, 25]. Most bLGG had a larger tumor volume than the non-butterfly ccLGG, and thus the patients with bLGG had a poorer survival. In addition, the bLGG had more subventricular zone involvement and higher probability of residual tumor cells on the edge of resection cavity which cannot be detected by iMRI. These factors were reported to be associated with patients’ poorer survival and multifocal recurrence of LGG [26, 27]. Our results indicate that the patient with bLGG can benefit from multimodal techniques.

The prognosis analysis showed that multimodal approach can both improve the PFS and OS of patients with ccLGG by univariate analysis. However, the multivariate analysis did not show the benefit of multimodal approach on survival. There may exist a collinearity issue between multimodal approach and other factors (for example EOR and KPS). Thus, the benefit of multimodal approach was instrumental rather than inherent to the technique, it improved the survival of ccLGG by the increment of EOR and preservation of neurological function. Many factors were associated with the PFS of ccLGG, including bLGG, tumor volume, site of CC invasion, volume of CC invasion, EOR, pathology, TMZ cycles, postoperative KPS on 3 months, MGMT methylation status. The tumor tended to be unresectable and may had lower EOR if it was the butterfly glioma, or had larger volume or involved corpus callosum, which was reported in previous studies [7, 28–30]. Thus, these factors influenced the PFS of ccLGG through decreasing the EOR. A meta-analysis demonstrated that postoperative radiotherapy and chemotherapy was not associated with the OS of LGG, however they can prolong the 5- and 10-year PFS [31]. Our results demonstrated postoperative radiotherapy was not associated with the survival of ccLGG, while more cycles of TMZ chemotherapy could benefit both PFS and OS of patients with ccLGG. Postoperative KPS represented the integrative neurological status of patients of ccLGG. The shorter PFS meant earlier recurrence of tumor which can lead to the worse neurological status of patients, so that the association of KPS with PFS can be explained. Four factors were identified to influence the OS of ccLGG significantly in our study. The EOR, TMZ cycles, and MGMT methylation were proved their positive influence on survival by previous studies, which was consistent with our findings. Interestingly site of CC invasion was another important factor that influence the survival of ccLGG. In this study, it was demonstrated that ccLGG involving splenium or both genu and body of corpus callosum had worse prognosis than ccLGG involving genu of CC alone. We thought that if the tumor involved splenium, body or more than two parts of CC, it was more difficult to be removed because it can be near the functional area, midline structures and important draining veins. Although astrocytoma had shorter median PFS and OS than oligodendroglioma in the multimodal group, the PFS and OS were both not significantly different among patients diagnosed either with astrocytoma or oligodendroglioma in the conventional group. pathology (astrocytoma versus oligodendroglioma) was not significantly associated with PFS or OS in the survival analysis. Because the lower EOR may cause this phenomenon that the impact of pathology on survival was eliminated in the conventional group. Thus, either astrocytoma or oligodendroglioma can achieve good prognosis similarly after the maximal safe resection and adjuvant therapy. As higher EOR independently predicts improved survival in IDH-mutant ccLGG, maximal safe resection should be suggested and achieved by appropriate means tailored to available resources, especially to patients with some type of ccLGG, such as bLGG, ccLGG of large tumor volume, ccLGG of splenium invasion or both genu and body invasion. The TMZ chemotherapy should be recommended to patients with high risks (age≧ 60 years, not gross total resected, tumor size > 6 cm in diameter, neurological defects before surgery). While whether postoperative radiotherapy should be performed should consider and balance many factors, for example, risks, benefit and tolerance of patients.

Our multimodal cohort achieved better outcomes than in previous studies of ccLGG (Table 5) [5, 6, 32]. Other large LGG cohorts were also compared with our ccLGG cohort (Table 5) [7, 33–49]. Previous studies of LGG reported the GTR rate of meta-analysis was 42.8% (95% CI [32.0–53.6%]) (Supplementary Fig. S3A). Meta-analysis of the 5-year survival was 83.7% (95% CI [77.7–89.6%]) (Supplementary Fig. S3B). Our ccLGG cohort of multimodal group achieved the GTR rate of 62.5%, which was higher than the 95% CI of GTR rate of previous studies of LGG. While our ccLGG cohort of multimodal group achieved the 5-year survival of 84.4%, which was within the 95% CI of 5-year survival of previous studies of LGG. The median PFS (78.5 months) of our ccLGG cohort was longer than those of most previous studies, but shorter than that of a study by Zeng (2021) [44]. Most previous studies cannot achieve median OS because the follow-up was not long enough. Our OS (106.1 months) was slightly shorter than those of 4 LGG previous studies because of the inherent characteristics of ccLGG, and was longer than that of Lote’s study (1997) [33–35, 43, 47]. The incidences of postoperative motor and language deficits were also within the range of previous studies. Furthermore, the incidences of cognitive deficit and other complications were lower in our ccLGG patients. Because of ccLGG with a tendency of local and multifocal recurrence compared with non-ccLGG, a higher EOR is necessary and can be achieved by surgery assisted by multimodal techniques. Thus, similar outcome can be achieved in patients with ccLGG as the patients with non-ccLGG by using multimodal techniques.

**Table 5:** Compared our multimodal group with ccLGG and LGG cohorts of previous studies

Study	Patients of surgery (N)	Included patients	100% GTR (*N* [%])	Median PFS (months)	Median OS (months)	5-year survival	Permanent motor deficit (%)	Permanent language deficit (%)	Permanent cognitive deficit (%)	Other complication rate (%)
Duffau et al., 2004 [32]	32	ccLGG	9 (28.1)		36.0	/	Total neurological deficits = 21.9%			/
Burks et al., 2016 [6]	40	Butterfly glioma (LGG = 13)	33 (82.5)	/	/	/	17.5%	/	/	22.5%
Chen et al., 2015 [5]	11	ccLGG	0 (0)	25.0	48.0	54.5%	/	/	/	/
Scerrati et al., 1996 [33]	131	LGG	76 (58.0)	/	144	97.1%	/	/	/	/
Lote et al., 1997 [34]	379	LGG	/	/	100.0	/	/	/	28.0%	/
Smith et al., 2008 [35]	216	LGG	75 (34.7)	66.0	121.2	85.8%	10.6%	5.6%	/	/
Shaw et al., 2008 [36]	362	LGG	111 (30.7)	58.8	NA (85.2)	74.3%	/	/	/	/
Jakola et al., 2012 [37]	87	LGG	/		NA (120)	74%	Total neurological deficits = 20.7%			8.0%
Ius et al., 2012 [38]	190	LGG	91 (47.9)	42.0	NA (155)	80%	/	/	/	/
Lima et al., 2015 [39]	21	LGG	14 (66.7)	22.5	/	/	0	0	/	/
Wijnenga et al., 2017 [40]	83	LGG	/	/	NA (252)	81.9%	/	/	/	/
Muto et al., 2018 [41]	39	LGG	18 (46.2)	/	/	/	7.7%	10.3%	25.6%	/
Ng et al., 2019 [42]	74	LGG	22 (29.7)	/	NA (240)	100%	/	/	/	/
Paľa et al., 2020 [43]	144	LGG	32 (22.2)	46.8	193.2	/	/	/	/	/
Zeng et al., 2021 [44]	75	LGG	59 (78.7)	80.0	/	72%	7.7%	3.8%	/	8.0%
Wang et al., 2022 [7]	138	LGG	63 (45.7)	60	/	/	/	/	/	/
Ius et al., 2022 [45]	267	LGG	159 (61.6)	/	87.6 (Median FU)	98.09%	3.1%	/	/	/
Lemaitre et al., 2022 [46]	157	LGG	44 (28.0)	/	/	82.2%	/	/	/	/
Sauvageot et al., 2023 [47]	108	LGG	/	/	138	80%	0.9%	/	/	4.6%
Bankole et al., 2023 [48]	61	LGG	/	/	/	94%	/	13.1%	3.28%	/
Duffau 2025 [49]	253	LGG	32 (12.7)	/	85.2 (mean FU)	80.2%	0%	0.39%	/	/
Present study	64	ccLGG	40 (62.5)	78.5	106.1	84.4%	4.7%	6.3%	9.4%	0%

*FU* follow-up, *NA* not available because less than 50% had progression or died at the end of the follow-up, the longest follow-up was provided in the parenthesis

## Limitations

To the authors’ knowledge, this study is the largest series of ccLGG resection. Due to the retrospective design, this study had some inherent bias, including selection bias, recall bias and attrition Bias. While baseline characteristics of groups were balanced, which demonstrated confounders were controlled well in this study. Enough possible prognostic factors of ccLGG were included to perform survival analysis, so that the selection bias was controlled within a reasonable range. Because of limited cases of IDH-mutant ccLGG, this study compared multimodal approach with only a subset of contemporary techniques (neuronavigation alone). Although this study demonstrated excellent outcomes with multimodal techniques, the comparative advantage over other resection-focused approaches remained uncertain given the retrospective design. Prospective randomized studies of large samples would be needed to definitively establish the specific value of iMRI and IONM in this population. In our previous study, the *PDGFRA* alterations were proved to have higher incidences in GBM of CC involvement and were associated with the poorer prognosis [50]. Detail molecular alterations cannot be explored in this study because of the limited cases of ccLGG had Next-Generation DNA Sequencing. The molecular alterations of ccLGG should be explored in further studies.

## Conclusion

The combined surgical approach assisted by multimodal techniques is useful to achieve maximal safe resection for ccLGG. Our findings underscore that multimodal surgical resection was associated with improved extent of resection and survival, highlighting the instrumental role of IONM and iMRI in optimizing the surgical management of IDH-mutant ccLGGs.

The EOR can be increased significantly in parallel with the preservation of neurological function and improvement of survival. Other than some treatment factors (EOR, TMZ cycles), some intrinsic characteristics of tumor were associated with the PFS or OS of ccLGG, including bLGG, tumor volume, site of CC invasion, CC invasion volume and MGMT methylation status. As higher EOR independently predicts improved survival in IDH-mutant ccLGG, maximal safe resection should be suggested and achieved by appropriate means tailored to available resources, especially to patients with some type of ccLGG, such as bLGG, ccLGG of large tumor volume, ccLGG of splenium invasion or both genu and body invasion and ccLGG of MGMT methylation. The postoperative chemotherapy should also be recommended to patients with high risks. While whether postoperative radiotherapy should be performed should consider and balance many factors, for example, risks, benefit, and tolerance of patients.

## Supplementary Information

Supplementary Material 1: Supplementary Figure S1. Postoperative MRI images corresponding to Fig. 2.

Supplementary Material 2: Supplementary Figure S2. Comparison of survival curves between oligodendroglioma and astrocytoma. In the multimodal group, median PFS 99.7 versus 63.7 months (A), median OS 108.6 versus 103.3 months (B). In the conventional group, median PFS 39.2 versus 64.6 months (C), median OS 78.5 versus 91.7 months (D).

Supplementary Material 3: Supplementary Figure S3. Forest plots of previous studies by meta-analysis. A: pooled GTR rate, B: pooled 5-year survival rate.

Supplementary Material 4: Supplementary Table S1. Comparison between the bLGG and non-butterfly ccLGG in the multimodal group.

## Data Availability

The reasonable requirement of original data and materials of this study can be obtained by contacting with corresponding authors.

## Abbreviations

AQ: Aphasia quotient
bLGG: Butterfly low-grade glioma
ccLGG: Low-grade glioma involving corpus callosum
EOR: Extent of resection
GTR: Gross total resection
HR: Hazard ratio
IDH: Isocitrate dehydrogenase
iMRI: Intraoperative MRI
IONM: Intraoperative neuromonitoring
KPS: Karnofsky Score
MGMT, O^6^: Methylguanine DNA methyltransferase
MoCA: Montreal Cognitive Assessment scale
OS: Overall survival
PFS: Progression-free survival
