# Supplementary figures and images for: Multimodal techniques for maximal safe resection of IDH-mutant low-grade glioma involving corpus callosum, a retrospective study and prognosis analysis
Source: Chin Neurosurg J. 2026 May 1;12:13. doi: 10.1186/s41016-026-00432-y (PMC13134284; doi:10.1186/s41016-026-00432-y)

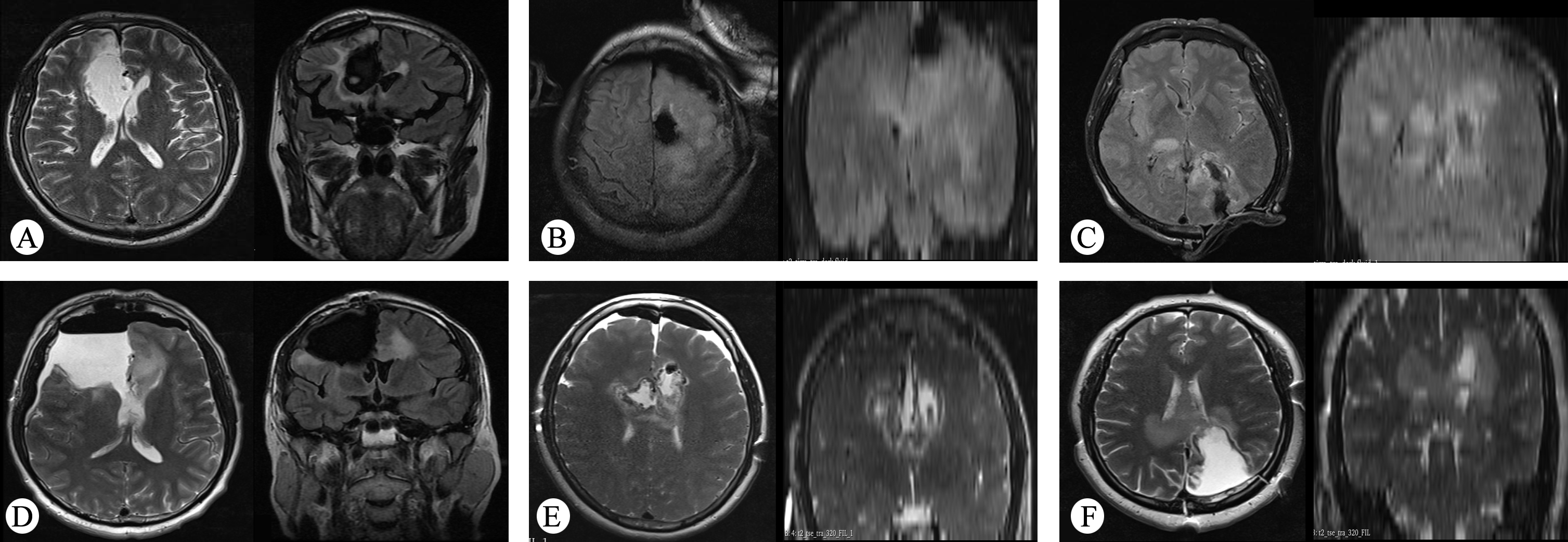

Supplement: Supplementary file 1 — Supplementary Material 1: Supplementary Figure S1. Postoperative MRI images corresponding to Fig. 2. [file 41016_2026_432_MOESM1_ESM.jpg]

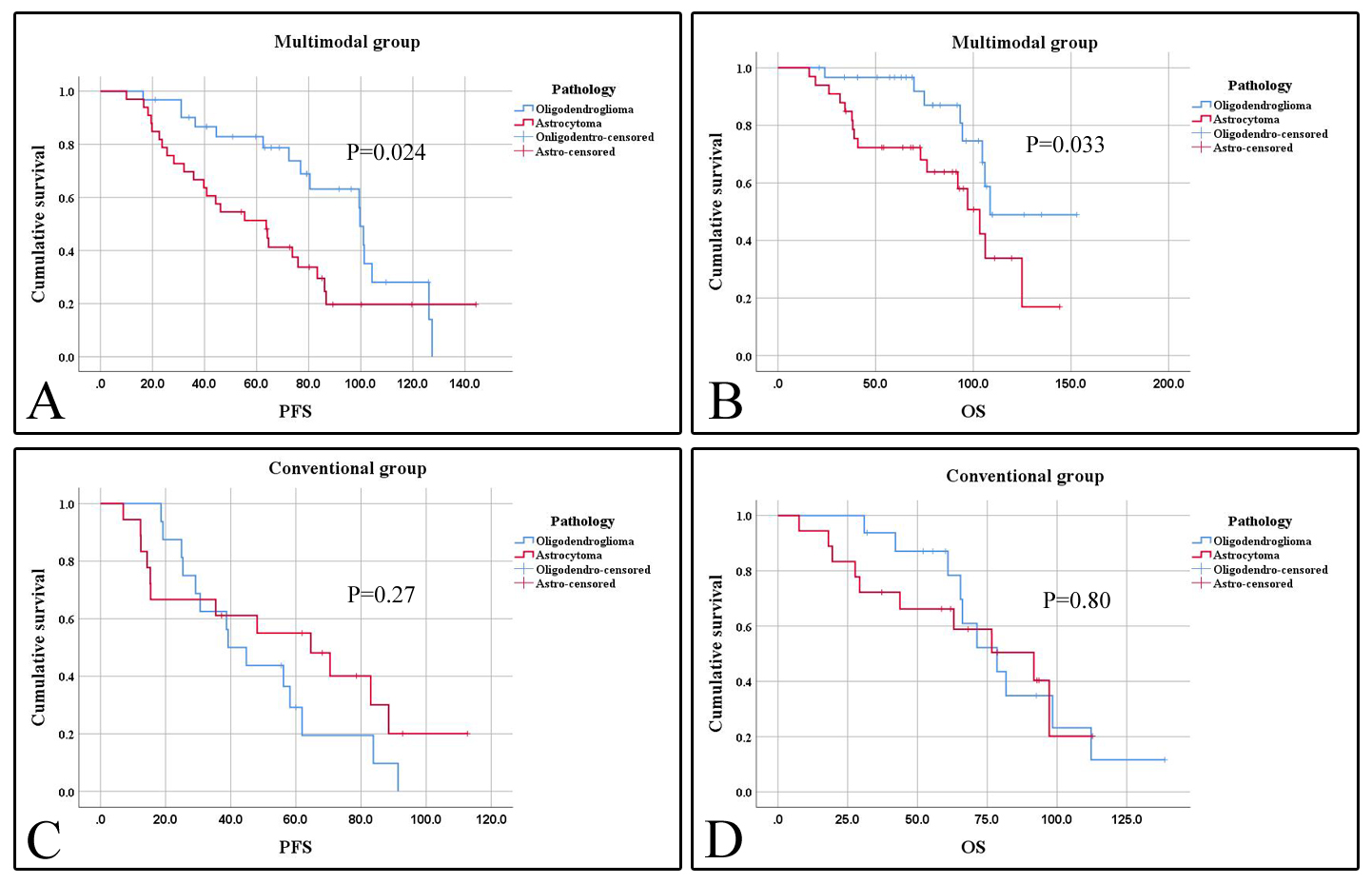

Supplement: Supplementary file 2 — Supplementary Material 2: Supplementary Figure S2. Comparison of survival curves between oligodendroglioma and astrocytoma. In the multimodal group, median PFS 99.7 versus 63.7 months (A), median OS 108.6 versus 103.3 months (B). In the conventional group, median PFS 39.2 versus 64.6 months (C), median OS 78.5 versus 91.7 months (D). [file 41016_2026_432_MOESM2_ESM.jpg]

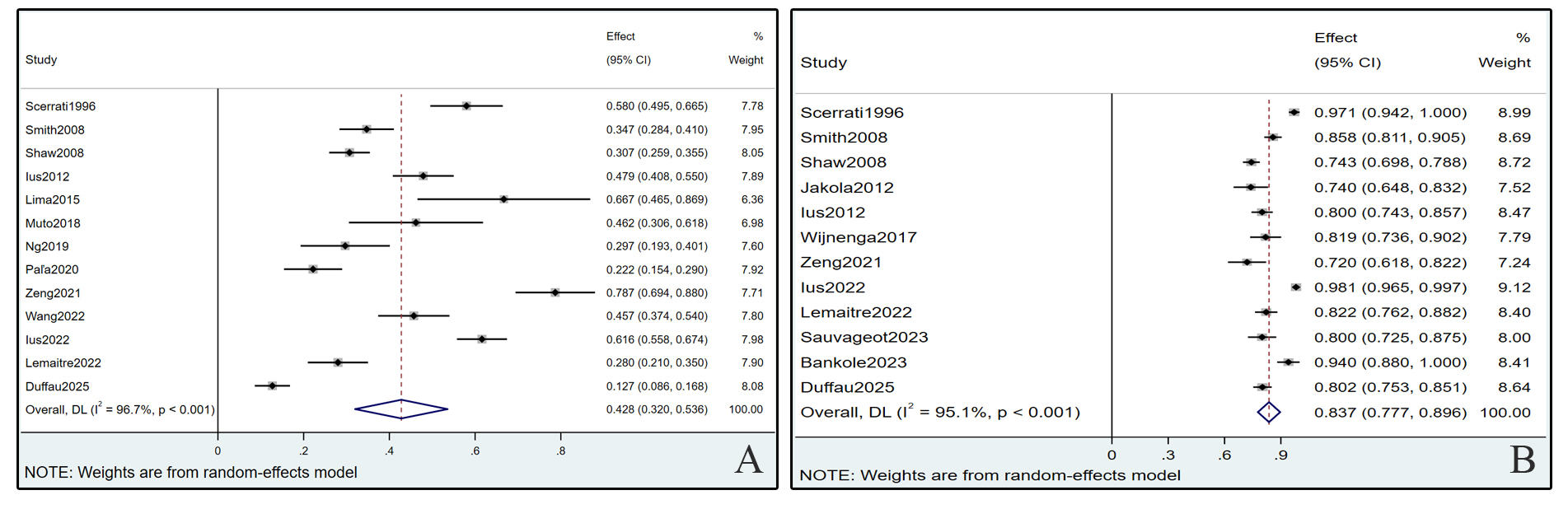

Supplement: Supplementary file 3 — Supplementary Material 3: Supplementary Figure S3. Forest plots of previous studies by meta-analysis. A: pooled GTR rate, B: pooled 5-year survival rate. [file 41016_2026_432_MOESM3_ESM.jpg]
