# Supplementary material for: Multimodal techniques for maximal safe resection of IDH-mutant low-grade glioma involving corpus callosum, a retrospective study and prognosis analysis
Source: Chin Neurosurg J. 2026 May 1;12:13. doi: 10.1186/s41016-026-00432-y (PMC13134284; doi:10.1186/s41016-026-00432-y)
Supplement: Supplementary file 4 — Supplementary Material 4: Supplementary Table S1. Comparison between the bLGG and non-butterfly ccLGG in the multimodal group. [file 41016_2026_432_MOESM4_ESM.docx]

Supplementary table 1. Comparison between the bLGG and non-butterfly ccLGG in the multimodal group

| Variables | bLGG  (N=24) | Non-butterfly ccLGG  (N=40) | P |
| --- | --- | --- | --- |
| Age (years, ‾x ± sd)† | 40.0±9.2 | 44.2±12.6 | 0.16 |
| Sex (N [%]) |  |  | 0.30 |
| Male | 10 (41.7) | 22 (55.0) |  |
| Female | 14 (58.3) | 18 (45.0) |  |
| Median Preop. KPS (IQR)‡ | 90 (72.5-90) | 80 (70-87.5) | 0.08 |
| Preop. Total tumor Vol (cm^3^, median [IQR])‡ | 70.01 (55.56-92.36) | 61.62 (40.27-89.28) | 0.46 |
| CC invasion Vol (cm^3^, median [IQR])‡ | 5.06 (4.12-7.02) | 4.26 (3.74-6.86) | 0.27 |
| Ratio of CC invasion/Total Vol (%, Median [IQR])‡ | 7.06 (5.96-10.49) | 7.31 (4.74-11.48) | 0.66 |
| High risks (N [%]) | 21 (87.5) | 32 (80.0) | 0.67 |
| *MGMT* methylation (N [%]) | 13 (54.2) | 29 (72.5) | 0.14 |
| Radiotherapy (N [%]) | 19 (79.2) | 28 (70.0) | 0.42 |
| TMZ chemotherapy (N [%]) | 23 (95.8) | 33 (82.5) | 0.24 |
| Median TMZ cycles (IQR)‡ | 6 (6-15.75) | 8.5 (6-15) | 0.91 |
| Median surgery time (IQR)‡ | 7.17 (6.69-7.5) | 7.09 (6.19-8.29) | 0.73 |
| Outcomes |  |  |  |
| Median length of hospital stay (IQR)‡ | 19 (15.25-22) | 18.5 (16-21.75) | 0.73 |
| Median EOR (% [IQR])‡ | 99.84 (94.49-100) | 100 (97.24-100) | 0.14 |
| Rate of GTR (N [%]) | 12 (50.0) | 28 (70.0) | 0.11 |
| Median KPS at discharge (IQR)‡ | 90 (72.5-100) | 80 (70-90) | 0.21 |
| Median KPS at 3 months (IQR)‡ | 90 (90-100) | 90 (72.5-90) | 0.06 |
| Median PFS in months (95%CI) | 44.3 (21.1-67.5) | 86.7 (59.8-113.6) | **0.004** |
| Median OS in months (95%CI) | 97.1 (63.4-130.8) | 108.6 (90.5-126.7) | **0.019** |
| 5-year survival (N [%]) | 16 (66.7) | 38 (95.0) | **0.008** |

IQR: Interquartile range, sd: standard deviation, †calculated by independent samples t test, ‡calculated by Mann-Whitney U-test.
